# Impact of Extraosseous Extramedullary Disease on Outcomes of Patients with Relapsed-Refractory Multiple Myeloma receiving Standard-of-Care Chimeric Antigen Receptor T-Cell Therapy

**DOI:** 10.1038/s41408-024-01068-w

**Published:** 2024-05-31

**Authors:** Danai Dima, Al-Ola Abdallah, James A. Davis, Hussein Awada, Utkarsh Goel, Aliya Rashid, Shaun DeJarnette, Faiz Anwer, Leyla Shune, Shahzad Raza, Zahra Mahmoudjafari, Louis Williams, Beth Faiman, Joseph P. McGuirk, Craig S. Sauter, Nausheen Ahmed, Jack Khouri, Hamza Hashmi

**Affiliations:** 1 Department of Hematology/Oncology, Cleveland Clinic, Taussig Cancer Institute, Cleveland, OH USA; 2 US Myeloma Innovations Research Collaborative (USMIRC), Westwood, KS USA; 3 Division of Hematologic Malignancies & Cellular Therapeutics, University of Kansas Medical Center, Westwood, KS USA; 4 Division of Hematology/Oncology, Medical University of South Carolina, Charleston, SC USA; 5 Memorial Sloan Kettering Cancer Center, New York, NY USA

**Keywords:** Myeloma, Risk factors

## Abstract

The presence of extramedullary disease (EMD) has been associated with poor outcomes in patients with relapsed-refractory multiple myeloma (RRMM). Herein, we report the outcomes of RRMM patients who were treated with standard-of-care (SOC) chimeric antigen receptor (CAR) T-cell therapy and had active extraosseous EMD before the infusion. Data were retrospectively collected from patients at three US institutions with the intent to receive SOC CAR T. Responses were assessed per the International Myeloma Working Group criteria. A total of 152 patients proceeded with infusion, of whom 47 (31%) had EMD (EMD group) and 105 (69%) did not (non-EMD group). Baseline patient characteristics were comparable between the two groups. The EMD group had a higher incidence of high-grade CRS, steroid and anakinra use, and thrombocytopenia on day +30 compared to the non-EMD group. In addition, the EMD group had an inferior overall response rate (58% vs 96%, *p* < 0.00001), median progression-free survival (PFS) (5.1 vs 12.4 months; *p* < 0.0001), and overall survival (OS) (12.2 vs 27.5 months; *p* = 0.00058) compared to the non-EMD group. We further subdivided the non-EMD patients into those with paramedullary disease (PMD-only group, *n* = 26 [17%]) and those with neither EMD nor PMD (bone marrow-contained group or BM-only group, *n* = 79 [52%]). Patients with PMD-only had similar median PFS (11.2 vs 13.6 months, *p* = 0.3798) and OS (not reached [NR] vs 27.5 months, *p* = 0.6446) compared to patients with BM-only disease. However, patients with EMD exhibited inferior median PFS (5.1 vs 13.6 months, *p* < 0.0001) and OS (12.2 vs 27.5, *p* = 0.0008) compared to patients in the BM-only group. Treatment with SOC CAR T yielded meaningful clinical outcomes in real-world RRMM patients with extraosseous EMD, though responses and survival outcomes were suboptimal compared to patients without EMD. The presence of only EMD but not PMD was associated with significantly worse survival outcomes following the CAR T infusion.

## Introduction

Extramedullary disease (EMD) represents an uncommon and aggressive manifestation of multiple myeloma (MM) [1, 2]. EMD is rarely seen in newly diagnosed MM, rather, it appears to evolve over time, with an incidence between 3 and 14% in relapsed-refractory MM (RRMM). EMD has previously been associated with adverse cytogenetic features and treatment-resistant disease [3]. While the current literature has identified EMD as a poor prognostic feature, at present, there are no consensus guidelines on how to treat EMD, given the relative lack of quality data [3, 4].

The recent introduction of novel agents has radically changed the treatment paradigm of RRMM [5]. Idecabtagene vicleucel (ide-cel) was the first chimeric antigen receptor (CAR) T-cell therapy product approved in March 2021 by the United States (US) Food and Drug Administration (FDA) for the treatment of RRMM after four or more prior lines of therapy, including a proteasome inhibitor, immunomodulator and anti-CD38 monoclonal antibody based on the results of the pivotal phase I/II KarMMa-1 trial [6]. This was followed by the approval of ciltacabtagene autoleucel (cilta-cel) in February 2022 based on the results of the phase I/II CARTITUDE-1 trial [7]. While the response rates and survival outcomes have been very promising, recently published prospective and real-world data suggested that the presence of EMD predicts early progression after CAR T-cell therapy [8–12].

The aim of this multicenter study was to perform an in-depth assessment of the outcomes of RRMM patients who were treated with commercial CAR T-cell therapy and had active extraosseous extramedullary disease before the infusion.

## Methods

### Study design and data collection

This is a multicenter retrospective analysis of adult patients with RRMM who received commercial anti-B-cell maturation antigen (BCMA) CAR T-cell therapy as per FDA label between 8/1/21 and 6/30/23. This study included three large academic centers in the U.S., part of the U.S. Myeloma Innovations Research Collaborative (USMIRC). All centers obtained institutional review board approval, which granted a waiver of consent, and the study was conducted in accordance with the Declaration of Helsinki. The data-cutoff date was December 15, 2023, for the safety and efficacy analyses. We previously reported on a cohort of 133 patients with RRMM who received ide-cel or cilta-cel in the real-world setting [13]. The patients in this study include an extended duration of follow-up for the first 133 patients infused, as well as 19 additional patients who received commercial CAR T since the last data cutoff.

Lymphodepleting chemotherapy with either cyclophosphamide 300 mg/m^2^ plus fludarabine 30 mg/m^2^ on days −5, −4, and −3 or bendamustine at 90 mg/m^2^ was administered on days −4, −3 prior to CAR T-cell infusion in the era of the national fludarabine shortage. The fludarabine dose was adjusted for creatinine clearance (CrCl) per institutional guidelines. Bridging therapy (when given) was administered ≥14 days prior to the initiation of lymphodepleting chemotherapy. Active EMD before CAR T in this study was defined as bone-independent (only) tumors of plasma cells growing at anatomical sites outside of the bone marrow detected within 30 days of CAR T-cell infusion. Bone-dependent and paraskeletal plasmacytomas (paramedullary disease) were not considered as EMD. However, knowing that paraskeletal plasmacytomas have been classified as EMD in some reported literature and to better understand the impact of ‘true EMD’ on outcomes post CAR T, we further subdivided patients without EMD into those with paramedullary disease (PMD)-only and those without any EMD or PMD. Visceral EMD was defined as EMD located in major internal organs, including but not limited to the liver, lungs, spleen, and pancreas (supplementary Table 1). High-risk cytogenetics were defined as the presence of deletion 17p, t(4;14), and/or t(14;16) on fluorescence in situ hybridization testing.

Cytokine release syndrome (CRS) and immune-effector cell-associated neurotoxicity syndrome (ICANS) were graded according to the American Society for Transplantation and Cellular Therapy (ASTCT) consensus criteria [14]. Hematologic toxicities were graded as per Common Terminology Criteria for Adverse Events (CTCAE), version 5.0 [15]. Infectious disease prophylaxis, use of growth colony-stimulating factors (GCSF), and management of CRS and ICANS were per institutional guidelines. While there were some differences based on institutional protocols, overall management guidelines were similar and in accordance with previously published guidelines. Responses to therapy were assessed using the International Myeloma Working Group (IMWG) criteria [16]. For patients with EMD, hematologic, radiographic, and combined hematologic and radiographic responses were evaluated. All patients with EMD who were alive at day +30 post infusion, except five, had at least one follow-up whole body imaging assessment with either PET/CT or MRI between day +30 and +90 after the infusion. However, assessments were not obtained at regular time intervals due to the retrospective nature of the study and the barriers encountered in the real-world setting with regard to imaging completion. Progression-free survival (PFS) was defined as the time from CAR T-cell infusion until disease progression or death from any cause, whichever occurred first. Overall survival (OS) was defined as the time from CAR T-cell infusion until death from any cause or last follow-up.

### Statistical analysis

Baseline patient and disease-related characteristics, safety, and efficacy outcomes were outlined with descriptive statistics. Differences between groups were evaluated using chi-squared or Fisher’s exact tests for categorical variables or Kruskal–Wallis rank-sum tests for continuous variables. For categorical variables such as overall response rate (ORR), and complete response (CR) or better (≥CR), CRS, and ICANS logistic regression analysis was used. The PFS and OS were estimated by the Kaplan–Meier method and were analyzed using the log-rank tests. For multivariable analysis, Cox proportional hazards models were used. Both univariable and multivariable analyses were performed to determine the association between patient and disease characteristics with CAR T outcomes. All statistical tests were two-sided, and *p*-values of less than 0.05 were considered statistically significant. All statistical analyses were conducted in R software, version 4.3.1.

## Results

### Baseline characteristics

As of December 15, 2023, 189 RRMM patients underwent leukapheresis with intent to manufacture commercial CAR T. A total of 152 patients proceeded with infusion, 14 patients did not receive CAR T because of disease progression/death, and 23 were pending infusion at data cut-off. Of the 152 patients who received CAR T and were included in this analysis, 108 (71%) received standard of care (SOC) ide-cel, and 44 (29%) received SOC cilta-cel. Forty-seven (31%) patients had active EMD before the CAR T-cell infusion. Of the remaining 105 (69%) patients (non-EMD group), 26 (17%) had PMD-alone, and 79 (52%) had neither EMD nor PMD (bone marrow-contained group or BM-only group). Patients had a median of 6 (range 4–15) prior lines of therapy, and 85% had triple-class refractory disease.

Baseline patient characteristics are presented in Table 1, stratified by the groups who had active EMD (*n* = 47) (EMD group) prior to infusion compared to those who did not (*n* = 105) (non-EMD group). The median age of the EMD group was 60 (range 43–78) years, 19% had an Eastern Cooperative Oncology Group (ECOG) performance status (PS) ≥ 2, 43% had R-ISS stage III disease prior to infusion, and 35% had high-risk cytogenetics. Thirty-six (77%) patients received ide-cel and 11 (23%) patients received cilta-cel in the EMD group. The median age for patients in the EMD group was 60 years, as compared to 65 years for the non-EMD group (*p* = 0.0125). Otherwise groups were well matched (Table 1).

**Table 1:** Baseline characteristics of patients infused with CAR T-cell therapy.

Characteristic	Patients with active EMD prior to infusion (*N* = 47)	Patients without EMD prior to infusion (*N* = 105)	*p* Value
Median age (range)	60 (43–78)	65 (41–81)	0.0125
Male gender, *n (%)*	26 (55)	56 (53)	0.8615
*Race/ethnicity, n (%)*			
White	39 (83)	84 (80)	0.8239
Black, Hispanic, or Other	8 (17)	21 (20)	
*ECOG PS, n (%)*			
0–1	38 (81)	94 (90)	0.1931
2–3	9 (19)	11 (10)	
*R-ISS stage, n (%)*			
Stage I	5/28 (18)	10/69 (15)	0.3621
Stage II	11/28 (39)	38/69 (55)	
Stage III	12/28 (43)	21/69 (30)	
High tumor marrow burden, *n (%)*	9/44 (21)	23/99 (23)	0.7130
High risk cytogenetics, *n (%)*	13/37 (35)	36/83 (43)	0.3965
deletion 17	7/37 (19)	21/83 (25)	
*t* (4;14)	4/37 (11)	12/83 (14)	
*t* (14;16)	3/37 (8)	3/83 (4)	
Plasma cell leukemia	1 (2)	7 (7)	0.2468
*Systemic bridging therapy*, *n* *(%)*	40 (85)	87 (83)	0.7296
Alkylator-based	24 (51)	34 (32)	
Selinexor-based	6 (13)	17 (16)	
PI-combos	5 (11)	16 (15)	
IMiD-combos	2 (4)	12 (12)	
Other	3 (6)	8 (8)	
Radiation prior to CAR T	17 (36)	5 (5)	<0.0001
Median prior lines of therapy (range)	6 (4–15)	6 (4–15)	0.5543
Prior autologous SCT, *n (%)*	40 (85)	77 (73)	0.1111
Prior allogeneic SCT, *n (%)*	2 (4)	3 (3)	0.6551
*Refractory status*, *n (%)*			
Triple refractory	41 (87)	88 (83)	0.5861
Penta refractory	19 (40)	39 (37)	0.7002
*Prior anti-BCMA therapy*, *n (%)*	8 (17)	17 (16)	0.8984
Belantamab mafodotin	4 (9)	13 (12)	
Teclistamab	3 (6)	0	
REGN5458	1 (2)	1 (1)	
SEA-BCMA	0	2 (2)	
Allogeneic CAR T	0	1 (1)	
*Type of product*, *n (%)*			
Idecabtagene vicleucel	36 (77)	72 (69)	0.3134
Ciltacabtagene autoleucel	11 (23)	33 (31)	

*BCMA* B-cell maturation antigen, *CAR T* chimeric antigen receptor T-cell therapy, *combos* combinations, *ECOG* Eastern Cooperative Oncology Group, *PS* performance status, *R-ISS* revised international staging system, *SCT* stem cell transplant.

Regarding the specific location of EMD before CAR T infusion, 25 (53%) out of 47 EMD patients had visceral EMD. The most commonly involved organs are described in Supplementary Table 1. Twenty-one (45%) of EMD patients had cutaneous involvement, and seven (15%) had lymph node involvement. Nine (19%) patients had only one EMD lesion, nine (19%) patients had two EMD lesions, and the remaining 29 (62%) patients had ≥3 EMD lesions. Notably, six (17%) patients had at least one lesion measuring greater than 5 cm in longest perpendicular diameter, and 17 (36%) patients had received radiation therapy for EMD prior to CAR T.

### Safety

Adverse events for both groups are summarized in Table 2. The median duration of hospitalization for the EMD group was 12 days (range 5–50), and 9 patients (19%) required intensive care unit stay during the hospitalization. The incidence of all grade CRS for the EMD group was 81%, with a median time to maximum grade CRS of four days. The incidence of all grade ICANS was 36% in the EMD group, with 21% having grade 1, 9% having grade 2, and 6% having grade 4 events. While five (11%) and three (3%) patients in the EMD and non-EMD groups experienced grade 3-4 CRS events, respectively, rates of tocilizumab use did not differ between the two groups. However, the use of steroids (40% vs 24%, p = 0.03) and anakinra (21% vs 3%, *p* = 0.0002) was more frequent in the EMD group. When focusing on the EMD group only, the rate of any grade (grade ≥ 3) CRS was 78% (3%) vs 91% (36%) in patients who received ide-cel versus cilta-cel, respectively. Likewise, the rate of any grade (grade ≥ 3) ICANS was 36% (0%) vs 36% (27%) in patients who received ide-cel versus cilta-cel, respectively.

**Table 2:** Adverse events in patients infused with CAR T-cell therapy.

Adverse event	Patients with active EMD	Patients without EMD	*p* Value
**CRS*, n (%)***	***N*** **=** **47**	***N*** = **105**	
Any grade (1–5)	38 (81)	82 (78)	0.7001
Grade 3–5	5 (11)	3 (3)	**0.0471**
**ICANS*, n (%)***	***N*** = **47**	***N*** = **105**	
Any grade (1–5)	17 (36)	26 (25)	0.1490
Grade 3–5	3 (6)	7 (7)	0.9480
Tocilizumab use, *n* (%)	32 (68)	62 (59)	0.2891
Steroids use, *n* (%)	19 (40)	25 (24)	**0.0368**
Anakinra use, *n* (%)	10 (21)	3 (3)	**0.0002**
Infections (all grade), *n* (%)	20 (43)	32 (30)	0.1469
G-CSF use, *n* (%)	25 (53)	54 (51)	0.8407
TPO agonist use, *n* (%)	18 (38)	16 (15)	**0.0208**
Stem cell boost, *n* (%)	11 (23)	4 (4)	**0.0002**
**Hematologic toxicity at Day 30 post CAR T infusion**	***N*** **=** **42**	***N*** **=** **105**	
*Neutropenia at day 30, n (%)*			
Any grade (1–5)	31 (74)	71 (68)	0.4619
Grade 3–5	20 (48)	38 (36)	0.2003
*Anemia at Day 30, n (%)*			
Any grade (1–5)	37 (88)	81 (77)	0.1317
Grade 3–5	9 (21)	16 (15)	0.3332
*Thrombocytopenia at Day 30, n (%)*			
Any grade (1–5)	40 (95)	78 (74)	**0.0006**
Grade 3–5	26 (62)	40 (38)	**0.0087**
**Hematologic Toxicity at Day 90 post CAR T infusion**	***N*** **=** **30**	***N*** **=** **96**	
*Neutropenia at Day 90*			
Any grade (1–5)	13 (43)	31 (32)	0.2681
Grade 3–5	3 (10)	14 (15)	0.5213
*Anemia at Day 90*			
Any grade (1–5)	20 (67)	46 (48)	0.0727
Grade 3–5	3 (30)	5 (5)	0.3475
*Thrombocytopenia at Day 90*			
Any grade (1–5)	20 (67)	45 (47)	0.0583
Grade 3–5	5 (17)	12 (13)	0.5598

*CRS* cytokine release syndrome, *ICANS* immune effector cell-associated neurotoxicity syndrome, *G-CSF* granulocyte-colony stimulating factor, *TPO* thrombopoietin receptor agonist.

Bold *p*-values indicates statistically significant.

At day +30 post CAR T infusion, grade ≥3 neutropenia, anemia, and thrombocytopenia were noted in 48%, 21%, and 62% of the patients in the EMD group, respectively. Compared to the non-EMD group, there was a higher incidence of any grade (95% vs 74%, *p* = 0.0006) and grade ≥3 thrombocytopenia (62% vs 38%, *p* = 0.0087) in the EMD group which translated to a higher rate of thrombopoietin agonist use (38% vs 15%; *p* = 0.02) for the EMD group. The incidence of documented infections (all grades) was similar between the two groups, 43% in the EMD group vs 30% in the non-EMD group.

### Treatment response

**Fig. 1:**
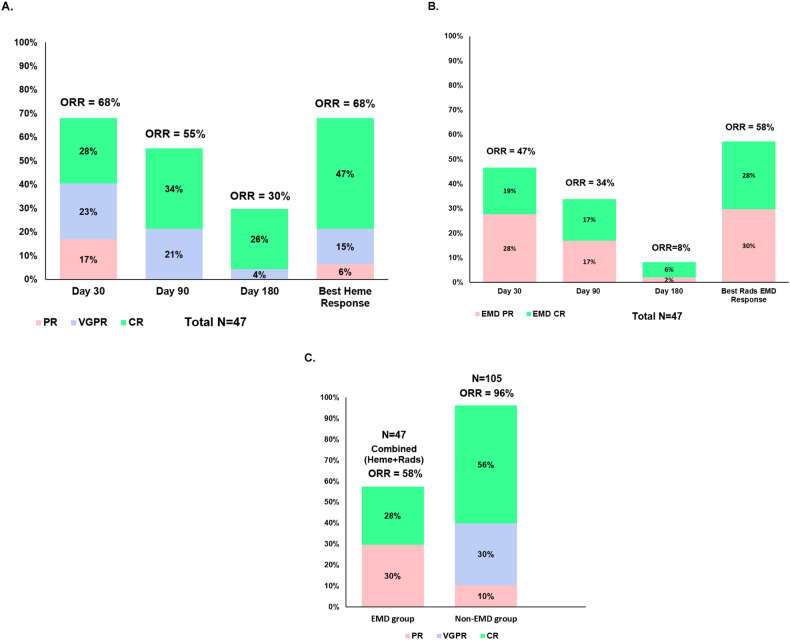
Response to CAR T-cell therapy. **A** Hematologic response rate for the EMD group. **B** Radiographic response rate for the EMD group. **C** Best overall tumor responses for the EMD (combined hematologic and radiographic response) and non-EMD groups. ORR overall response rate, CR complete response, VGPR very good partial response, PR partial response, heme hematologic, rads radiographic. * For response assessment at any time point, patients with missing data for rads or heme response, as well as deaths from toxicities/unrelated causes were considered as non-responders. Patients who died within the first 30 days post infusion prior to response assessment because of toxicities were also considered as non-responders.

The hematologic, radiographic, and combined hematologic and radiographic response rates for patients in the EMD group are shown in Fig. 1. Five out of the 47 patients were non-evaluable for combined overall response assessment since radiographic imaging was not obtained post infusion; these were considered as non-responders in the combined overall response analysis. In addition, patients who died within the first 30 days post infusion prior to response assessment because of toxicity were included in the response analysis and were considered as non-responders as well. Further details regarding hematologic and radiographic responses at days +30, +90, and +180 post CAR T for the EMD group are shown in Fig. 1A, B. For patients with EMD, the best combined ORR for those who received ide-cel (*n* = 36) and cilta-cel (*n* = 11) were 61% and 46%, respectively. Notably, four patients with EMD who received cilta-cel died from CAR T-related toxicities < 30 days post infusion, whereas none of the EMD patients treated with ide-cel died from toxicity < 30 days post infusion. Overall, patients with EMD had a lower combined ORR (58% vs 96%, *p* < 0.00001) and response of ≥CR (28% vs 56%, *p* = 0.002) compared to the non-EMD group (Fig. 1C).

When focusing on the EMD group, we explored EMD specific factors associated with radiographic response. On univariate analysis, we did not find any significant association between radiographic response rate and EMD characteristics, including number of lesions, size of lesions, presence of visceral EMD, and radiation prior to infusion.

### Survival outcomes

**Fig. 2:**
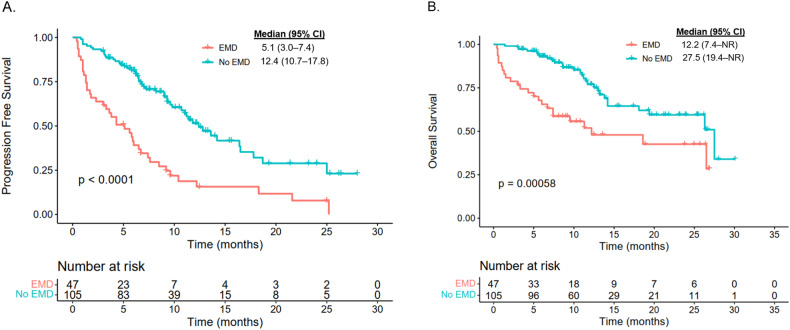
Survival outcomes of patients with EMD versus no EMD. **A** Progression-free survival. **B** Overall survival.

**Fig. 3:**
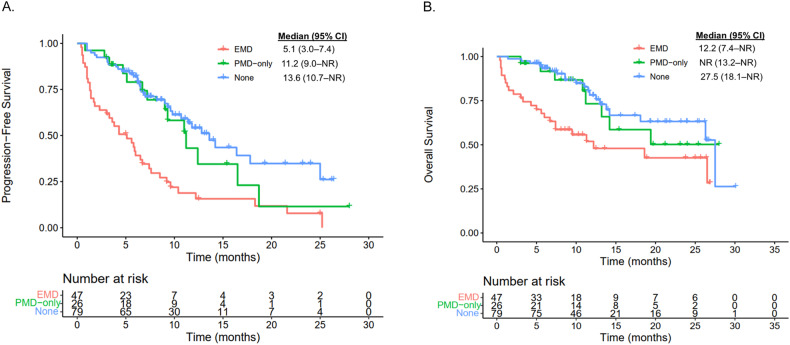
Survival outcomes of patients with EMD versus PMD-only versus no EMD/PMD (None). **A** Progression-free survival. **B** Overall survival.

The median duration of follow-up was 12.5 (IQR: 9.2, 25) months for the EMD group and 12.6 (IQR: 7.6, 23.1) months for the non-EMD group. Patients in the EMD group had an inferior median PFS compared to the non-EMD group (5.1 months vs 12.4 months; *p* < 0.0001; Fig. 2A). Similarly, patients in the EMD group had an inferior median OS compared to patients in the non-EMD group (12.2 vs 27.5 months; *p* = 0.00058; Fig. 2B). To better understand the impact of true EMD on efficacy post CAR T-cell therapy, we examined PFS and OS between the EMD, PMD-only, and BM-only groups. Patients with PMD-only disease had similar median PFS (11.2 vs 13.6 months, *p* = 0.3798; Fig. 3A) and OS (not reached [NR] vs 27.5 months, *p* = 0.6446; Fig. 3B) compared to patients with BM-only disease. However, patients in the EMD group exhibited significantly inferior median PFS (5.1 vs 13.6 months, *p* < 0.0001; Fig. 3A) and OS (12.2 vs 27.5, *p* = 0.0008; Fig. 3B) compared to patients in the BM-only group.

Multivariable analysis of the entire patient cohort identified active EMD before CAR T as an independent predictor for both inferior PFS (HR, 2.12; 95% CI, 1.26–3.6; *p* = 0.005) and OS (HR, 2.09; 95% CI 1.06–4.12; *p* = 0.033) (Table 3). In addition to active EMD, high-risk cytogenetics, prior anti-BCMA therapy, receipt of ide-cel product and high baseline ferritin were independently associated with worse PFS (Table 3); while active EMD and ECOG PS ≥ 2 were associated with worse OS. In the EMD cohort only, multivariate analysis identified ECOG PS ≥ 2, four prior lines of therapy, prior anti-BCMA therapy exposure, absence of radiation therapy prior to infusion, and lesion size of >5 cm to be associated with worse PFS (Table 4).

**Table 3:** Multivariable analysis for the association of selected patient and disease characteristics with PFS and OS for entire patient population.

	PFS			OS		
Variables	*N* (event *N*)	HR (95% CI)	*P*-value	*N* (event N)	HR (95% CI)	*P*-value
*Active EMD*			**0.005**			**0.033**
No	105 (49)	1.00 (reference)		105 (49)	1.00 (reference)	
Yes	47 (40)	2.12 (1.26–3.6)		47 (40)	2.09 (1.06–4.12)	
*Age*			0.353			0.136
≤65	87 (57)	1.00 (reference)		87 (57)	1. 00 (reference)	
>65	65 (43)	0.79 (0.48–1.3)		65 (43)	0.59 (0.30–1.18)	
*ECOG PS*			0.483			**0.024**
2–4	20 (14)	1.00 (reference)		20 (14)	1.00 (reference)	
0–1	132 (75)	0.75 (0.33–1.7)		132 (75)	0.35 (0.14–0.87)	
*Cytogenetics*			**0.039**			0.16
Standard risk	71 (59)	1.00 (reference)		71 (59)	1.00 (reference)	
High risk	49 (41)	1.77 (1.03–3.0)		49 (41)	1.60 (0.83–3.08)	
*Penta-refractory status*			0.099			0.509
No	94 (62)	1.00 (reference)		94 (62)	1.00 (reference)	
Yes	58 (38)	1.60 (0.92–2.8)		58 (38)	0.77 (0.36–1.65)	
*Prior BCMA therapy*			**<0.001**			0.149
No	127 (84)	1.00 (reference)		127 (84)	1.00 (reference)	
Yes	25 (16)	2.76 (1.52–5.0)		25 (16)	1.17 (0.83–3.55)	
*CAR T product*			**0.043**			0.829
Cilta-cel	44 (29)	1.00 (reference)		44 (29)	1.00 (reference)	
Ide-cel	108 (81)	2.19 (1.02–4.7)		108 (81)	0.91 (0.38–2.15)	
*Baseline CRP*			0.507			0.774
High	55 (36)	1.00 (reference)		55 (36)	1.00 (reference)	
Low	97 (64)	1.20 (0.7–2.0)		97 (64)	0.9 (0.46–1.79)	
*Baseline ferritin*			**0.049**			
High	90 (59)	1.00 (reference)		90 (59)	1.00 (reference)	0.106
Low	62 (41)	0.52 (0.28–1.0)		62 (41)	0.50 (0.22–1.16)	

*BCMA* B-cell maturation antigen, *CRP* C-reactive protein, *ECOG PS* Eastern Cooperative Oncology Group performance status, *EMD* extramedullary disease, *OS* overall survival, *PFS* progression free survival.

Bold *P*-values indicates statistically significant.

**Table 4:** Univariate and multivariate Cox Model for characteristics associated with progression-free survival in the EMD group only.

Variables	Progression-free survival			
	Univariate analysis		Multivariable analysis	
	HR (95% CI)	*p*-Value	HR (95% CI)	*p*-Value
*Age*				
<65 years	Ref.			
≥65 years	1.05 (0.53–2.075)	0.889		
*ECOG PS*				
0-1	Ref.		Ref.	
≥2	3.36 (1.50–7.52)	**0.0032**	3.90 (1.54–9.87)	**0.004**
*R-ISS*				
I	Ref.			
II	2.33 (0.66–8.14)	0.185		
III	0.68 (0.20–2.34)	0.547		
*Cytogenetics by FISH*				
Standard risk	Ref.			
High risk	1.46 (0.68–3.11)	0.322		
*Bone marrow plasma cell burden*				
Low	Ref.			
High (>50% plasma cells)	0.77 (0.33–1.81)	0.559		
*Bridging therapy*				
No	Ref.			
Yes	1.03 (0.43–2.46)	0.947		
*Number of prior lines*				
4	Ref.		Ref.	
>4	0.43 (0.19–1.009)	0.052	**0.22 (0.08–0.58)**	**0.021**
*Penta-refractory*				
No	Ref.		Ref.	
Yes	**1.96 (1.02–3.76)**	**0.042**	1.91 (0.92–3.97)	0.08
*Prior anti-BCMA therapy*				
No	Ref.		Ref.	
Yes	2.14 (0.95–4.80)	0.065	**7.72 (2.73–21.8)**	**0.0001**
*CAR T-cell product*				
Ide-cel	Ref.			
Cilta-cel	0.81 (0.33–1.95)	0.646		
*Baseline CRP*				
Low	Ref.			
High	1.41 (0.74–2.67)	0.291		
*Baseline ferritin*				
Low	Ref.			
High	1.45 (0.68–3.08)	0.327		
*Number of EMD lesions*				
1–2 lesions	Ref.			
3 or more lesions	1.14 (0.59–2.22)	0.64		
*Presence of visceral EMD*				
No	Ref.			
Yes	1.34 (0.71–2.54)	0.359		
*Radiation therapy prior to CAR T*				
No	Ref.		Ref.	
Yes	**0.48 (0.24–0.95)**	**0.037**	**0.39 (0.18–0.88)**	**0.023**
*Lesion size*				
Lesion size ≤5 cm	Ref.		Ref.	
Lesion size >5 cm	**3.29 (1.32**–**8.51)**	**0.0102**	**6.44 (2.14–19.33)**	**0.0008**

*EMD* extramedullary disease, *ECOG PS* Eastern Cooperative Oncology Group performance status, *R-ISS* revised international staging system, *FISH* fluorescence in-situ hybridization, *CRP* C-reactive protein.

Bold *p*-values indicates statistically significant.

Of the 47 patients in the EMD group, 32 (68%) experienced disease relapse, including 12 (25.5%) who relapsed with EMD, 8 (17%) who relapsed biochemically, and 12 (25.5%) who relapsed with both EMD and biochemically. EMD at relapse was present in the same site as prior to CAR T, in a new site, and both in the same plus new sites in seven (15%), nine (19%), and eight (17%) patients, respectively. At data cutoff, 24 (51%) out of the 47 patients in the EMD group had died; 14 (30%) from progressive myeloma; four (8.5%) from CRS/hemophagocytic lymphohistiocytosis, two (4%) from infection/sepsis and four (8.5%) from unrelated causes. Among the 105 patients in the non-EMD group, 28 (27%) patients had died at data cut-off; 24 (23%) from myeloma progression, two (2%) from infection/sepsis, one (1%) from delayed neurotoxicity, and one (1%) from unrelated causes.

## Discussion

This large multicenter real-world analysis delineates clinical outcomes of RRMM patients with active EMD treated with SOC CAR T-cell therapy. Based on our results, patients with active EMD before the CAR T infusion had inferior efficacy and survival outcomes, including ORR, ≥CR rate, PFS, and OS, compared to patients without active EMD. These findings highlight the presence of EMD as a poor prognostic factor for patients proceeding to CAR T. Notably, our definition of EMD was strict and only included lesions growing in anatomical sites outside of the bone marrow that had no contact with bony structures.

With an improvement in OS in the era of novel therapeutics, there is an increasing incidence of EMD for MM, especially at the time of relapse. The underlying pathophysiology is complex and may be explained by systemic inflammation and a more suppressive tumor microenvironment associated with large and highly avid tumors on positron emission tomography. Tumor-driven inhibition of T-cell function, whether it is T cells collected for manufacture or the CAR T-cells themselves after the infusion, adds to the effector target disparity because large tumors, as seen in EMD, require the highest expansion of CAR T for deep and durable responses [17].

Prospective data from the KarMMa-1 trial, reported that the high ORR and ≥CR rate of ide-cel was maintained in the subgroup of EMD patients, however, the rate of EMD was only 16% and EMD definition included contiguous bone lesions [18]. Similarly, the rate of EMD was low at 13% in the CARTITUDE-1 trial, and included both bone dependent and independent plasmacytomas. In the latter, the presence of plasmacytomas was associated with shorter PFS and OS [10]. Other early phase I/II trials of BCMA-directed CAR T products have also suggested that patients with EMD have inferior survival outcomes, however, sample sizes were limited and no extensive subgroups analyses were done [9, 19–21]. In a large real-world experience patients with EMD had a trend for inferior ORR and PFS to SOC ide-cel [22]; however, patients with prior history of EMD were grouped together with those who had active EMD prior to infusion. A recent retrospective analysis of 134 patients treated with CAR T reported findings similar to ours when categorizing the patients into EMD, PMD-alone, and BM-only disease groups [23]. However, the median PFS and OS of the EMD patients were double compared to those observed in our study [23]. This could be attributed to the inclusion of less heavily pre-treated trial patients and the use of CAR T products targeting other antigens beyond BCMA. In contrast, our study was a real-world experience with SOC anti-BCMA CAR T-cell therapy.

Apart from CAR T-cell therapy, other types of novel anti-BCMA therapies, such as bispecific T-cell engager antibodies, have also shown worse outcomes in the subset of patients with EMD. Teclistamab led to worse ORR in patients with EMD (36% vs 69%) than those without it in the pivotal phase II MajesTec-1 study (165 patients, 17% with EMD). This was confirmed in a recent real-world study of 106 RRMM patients treated with teclistamab where extra-osseous EMD was an independent factor predicting inferior ORR and PFS [24]. Elranatamab, another BCMA-directed bispecific T-cell agent, also led to lower ORR and duration of response in the EMD patients in the phase II MagnetisMM-3 trial [25].

While our study confirmed the previously reported negative prognostic impact of EMD, high risk cytogenetics, prior anti-BCMA therapy exposure, and elevated baseline ferritin on survival outcomes [26], it also highlighted risk factors within the EMD population that were associated with inferior outcomes. Worse ECOG PS at time of CAR T is often times a reflection of the negative impact of a more aggressive disease biology on patient’s physical fitness and organ reserve [27]. Similarly, prior BCMA-directed therapy has been established as a predictor of early progression and lack of response to CAR T [28], and this finding does support the use of CAR T in the earlier lines of therapy where patients are more likely to be BCMA-naïve. Patients who received >4 prior lines of therapy had better PFS, most likely due to inadvertently selecting for indolent disease biology in long term survivors. Previous reports have shown that bridging radiotherapy prior to anti-BCMA CAR T is feasible and safe [29]. Our study highlighted that patients who received radiation therapy prior to CAR T had better outcomes compared to those who did not. While this indeed underscores the need for more effective bridging therapy, including radiotherapy, for maximum debulking prior to CAR T infusion, this may not be attainable in many patients. Similarly, EMD lesions with size >5 cm were associated with inferior PFS, likely related to the augmented inflammatory state and inhibitory tumor microenvironment of larger sized EMD [30]. Based on our results, receipt of cilta-cel was an independent predictor for better PFS in the entire cohort, however, our sample size was limited thus no definite conclusions can be drawn. Larger studies comparing the two products are needed.

Lastly, our findings showed that, in most cases, relapse after CAR T for the EMD patients occurred radiographically (sometimes with concurrent biochemical relapse) rather than just biochemically. This suggests that EMD patients might benefit from more frequent routine imaging post CAR T to monitor closely for new, recurrent, or growing EMD lesions, as these may be asymptomatic in the early stages of progression or even precede biochemical relapse. Detecting EMD relapse early may allow for prompt intervention in this high-risk group of patients. Moreover, in the majority of patients, prior location of EMD remained the predominant site of post CAR T relapse, raising the possibility of EMD as site of immunological sanctuary susceptible to tumor relapse, which aligns with previously published data [31]. Investigating the mechanisms of CAR T-cell trafficking, surveillance, and senescence in patients with EMD will be essential to optimizing responses to CAR T cells and may identify factors leading to the rare responses to therapy, as seen in our study.

From a toxicity perspective, patients with active EMD in our study experienced more high-grade CRS, which could be related to higher disease burden and more profound inflammatory state contributed by EMD [17]. This likely explains the more frequent use of steroids and anakinra in the EMD group. Notably, four (8.5%) of the EMD patients, in addition to CRS, also developed HLH, the etiology of which was unclear. However, it could be partially related to the underlying poor performance status of these patients, EMD and medullary disease burden and/or concomitant infections (one patients had candida fungemia). The inflammatory state and suppressive tumor microenvironment associated with large and metabolically active tumors can explain the potential impact on hematopoiesis and platelet count recovery, necessitating more frequent use of thrombopoietin agonist and stem cell boost for EMD patients.

Limitations of our study include its retrospective design and limited sample size. While there was heterogeneity in institutional practices for toxicity management, this analysis is reflective of the variability seen in real-world practice patterns. Response assessment was per investigator discretion, and there was no independent review committee. In addition, due to the retrospective nature of the study, comprehensive imaging at regular time intervals and bone marrow-based MRD assessment were not always obtained. Overall, despite these limitations, our data represents a real-world population and is a true reflection of the real-life management of these patients. This study does allow us to identify patients more applicable for post CAR T interventions, including close surveillance with routine imaging for relapse, initiation of salvage therapy at the earliest signs of disease progression and consideration of maintenance therapy post CAR T in future clinical trials.

## Conclusion

In summary, this large multicenter retrospective study delineates real-world outcomes of SOC CAR T-cell therapy in RRMM patients with extraosseous EMD before the infusion. Based on our results, patients with EMD experienced more severe CRS and early thrombocytopenia. In addition, they had lower responses to commercial CAR T and experienced shorter PFS and OS compared to patients without EMD. Only the presence of EMD but not PMD was associated with significantly worse survival outcomes. These findings highlight the presence of active extraosseous EMD as a risk factor for inferior outcomes after SOC CAR T-cell therapy.

Despite the inferior outcomes compared to patients without EMD, CAR T still yielded meaningful clinical responses. Therefore, it should be considered as a treatment approach in EMD patients with good performance status, especially in the absence of more efficacious therapies or lack of other treatment options, including refractoriness to all traditional plasma-cell directed agents. Optimization with strategies targeting tumor shrinkage, such as chemotherapy or radiation before the infusion, may be advisable in an effort to extend the disease-free interval. Real-world data on outcomes of other recently approved novel agents, such as bispecific antibodies, in patients with active extraosseous EMD are also eagerly awaited.

### Supplementary information

Supplementary Material

## Data Availability

Data is available upon request.
