# Supplementary material for: Impact of Extraosseous Extramedullary Disease on Outcomes of Patients with Relapsed-Refractory Multiple Myeloma receiving Standard-of-Care Chimeric Antigen Receptor T-Cell Therapy
Source: Blood Cancer J. 2024 May 31;14(1):90. doi: 10.1038/s41408-024-01068-w (PMC11143360; doi:10.1038/s41408-024-01068-w)
Supplement: Supplementary file 1 — Supplementary Material [file 41408_2024_1068_MOESM1_ESM.docx]

**Supplemental Table 1.** Sites of extramedullary disease prior to CAR T-cell infusion.

| **Site of EMD** | **Patients with EMD**  **N=47 (100%)** |
| --- | --- |
| Visceral EMD |  |
| Lung | 8 (17%) |
| Liver | 8 (17%) |
| Pancreas | 3 (6%) |
| Spleen | 1 (2%) |
| Kidney | 1 (2%) |
| Testis | 1 (2%) |
| Colon | 1 (2%) |
| Pleura | 1 (2%) |
| Central nervous system | 1 (2%) |
| Soft Tissue | 27 (57%) |
| Lymph Note | 21 (45%) |
| Cutaneous | 7 (15%) |
| Breast | 2 (4%) |
| Orbit | 3 (6%) |
| Peritoneal involvement | 3 (6%) |
| Parotid Gland | 1 (2%) |

**Supplementary Table 2.** Univariate Analysis of the Entire Patient Population for Characteristics Associated with Progression Free Survival and Overall Survival.

|  | **Progression-Free Survival** | | **Overall Survival** | |
| --- | --- | --- | --- | --- |
| **Variable** | **HR (95% CI)** | **p-value** | **HR (95% CI)** | **p-value** |
| **Active EMD** |  |  |  |  |
| No | 1.00 (Ref) |  | 1.00 (Ref) |  |
| Yes | 2.892 (1.890 - 4.398) | <0.0001 | 2.546 (1.457 - 4.422) | 0.0009 |
| **Age** |  |  |  |  |
| >65 | 1.00 (Ref) |  | 1.00 (Ref) |  |
| ≥65 years | 0.70 (0.45 - 1.07) | 0.103 | 0.56 (0.31 - 1.004) | 0.051 |
| **Gender** |  |  |  |  |
| Female | 1.00 (Ref) |  | 1.00 (Ref) |  |
| Male | 1.075 (0.7085 - 1.635) | 0.7350 | 1.413 (0.8160 - 2.495) | 0.2225 |
| **ECOG PS** |  |  |  |  |
| 0-1 | 1.00 (Ref) |  | 1.00 (Ref) |  |
| ≥2 | 2.27 (1.27 - 4.058) | 0.0053 | 3.57 (1.85 - 6.90) | 0.0001 |
| **R-ISS** |  |  |  |  |
| 1 | 1.00 (Ref) |  | 1.00 (Ref) |  |
| 2 | 1.237 (0.5722 - 3.077) | 0.6145 | 1.703 (0.5774 - 7.265) | 0.3929 |
| 3 | 1.213 (0.5402 - 3.083) | 0.6592 | 1.651 (0.5290 - 7.223) | 0.4355 |
| **Marrow Burden, ≥50% plasma cells** |  |  |  |  |
| No | 1.00 (Ref) |  | 1.00 (Ref) |  |
| Yes | 1.313 (0.7886 - 2.108) | 0.2746 | 1.417 (0.7354 - 2.585) | 0.2735 |
| **Plasma Cell Leukemia** |  |  |  |  |
| No | 1.00 (Ref) |  | 1.00 (Ref) |  |
| Yes | 1.693 (0.6564 - 3.579) | 0.2156 | 2.315 (0.7974 - 5.356) | 0.0775 |
| **Presence of Amyloidosis** |  |  |  |  |
| No | 1.00 (Ref) |  | 1.00 (Ref) |  |
| Yes | 0.7074 (0.2157 - 1.701) | 0.4998 | 1.263 (Undefined) | 0.9999 |
| **Cytogenetics by FISH** |  |  |  |  |
| Low Risk | 1.00 (Ref) |  | 1.00 (Ref) |  |
| High Risk | 1.610 (0.9902 - 2.602) | 0.0521 | 1.741 (0.9361 - 3.266) | 0.0796 |
| **Receipt of Bridging therapy** |  |  |  |  |
| No | 1.00 (Ref) |  | 1.00 (Ref) |  |
| Yes | 1.339 (0.7763 - 2.487) | 0.3223 | 1.276 (0.6118 - 3.108) | 0.5505 |
| **Prior Autologous SCT** |  |  |  |  |
| No | 1.00 (Ref) |  | 1.00 (Ref) |  |
| Yes | 1.099 (0.6676 - 1.906) | 0.7231 | 1.077 (0.5605 - 2.281) | 0.8337 |
| **Prior Allogeneic SCT** |  |  |  |  |
| No | 1.00 (Ref) |  | 1.00 (Ref) |  |
| Yes | 1.198 (0.4967 - 3.937) | 0.7253 | 1.510 (0.4648 - 9.269) | 0.5694 |
| **Double refractory status** |  |  |  |  |
| No | 1.00 (Ref) |  | 1.00 (Ref) |  |
| Yes | 1.113 (0.6563 - 2.019) | 0.7069 | 0.9286 (0.4841 - 1.964) | 0.8340 |
| **Triple refractory status** |  |  |  |  |
| No | 1.00 (Ref) |  | 1.00 (Ref) |  |
| Yes | 1.484 (0.8056 - 3.059) | 0.2409 | 1.074 (0.5160 - 2.614) | 0.8601 |
| Penta refractory status |  |  |  |  |
| No | 1.00 (Ref) |  | 1.00 (Ref) |  |
| Yes | 1.915 (1.245 - 2.939) | 0.0029 | 1.259 (0.7172 - 2.176) | 0.4131 |
| **Prior anti-BCMA therapy** |  |  |  |  |
| No | 1.00 (Ref) |  | 1.00 (Ref) |  |
| Yes | 2.258 (1.345 - 3.641) | 0.0013 | 1.372 (0.6869 - 2.542) | 0.3390 |
| **CAR T Product** |  |  |  |  |
| Cilta-cel | 1.00 (Ref) |  | 1.00 (Ref) |  |
| Ide-cel | 3.033 (1.598 - 6.522) | 0.0018 | 0.7812 (0.3348 - 1.612) | 0.5325 |
| **Disease status** |  |  |  |  |
| Relapsed | 1.00 (Ref) |  | 1.00 (Ref) |  |
| Refractory | 1.16 (0.75 - 1.79) | 0.486 | 1.09 (0.62 - 1.93) | 0.742 |
| **Number of prior lines of therapy** |  |  |  |  |
| 4 | 1.00 (Ref) |  |  |  |
| >4 | 0.9683 (0.5775 - 1.727) | 0.9077 | 1.120 (0.5532 - 2.581) | 0.7704 |
| **Number of prior lines of therapy** | 1.074 (0.9814 - 1.168) | 0.1078 | 1.034 (0.9262 - 1.145) | 0.5295 |
| **Creatinine Clearance** |  |  |  |  |
| ≥45 mL/min | 1.00 (Ref) |  | 1.00 (Ref) |  |
| <45 mL/min | 0.9309 (0.4512 - 1.717) | 0.8319 | 1.206 (0.4945 - 2.518) | 0.6466 |
| **LVEF** |  |  |  |  |
| ≥45 % | 1.00 (Ref) |  | 1.00 (Ref) |  |
| <45% | 1.110 (0.2716 - 2.984) | 0.8592 | 2.233 (0.5386 - 6.184) | 0.1813 |
| **Baseline CRP Ferritin ≥ULN at LD** |  |  |  |  |
| No | 1.00 (Ref) |  | 1.00 (Ref) |  |
| Yes | 1.636 (1.076 - 2.485) | 0.0208 | 2.021 (1.170 - 3.516) | 0.0117 |
| **Baseline Ferritin ≥ULN at LD** |  |  |  |  |
| No | 1.00 (Ref) |  | 1.00 (Ref) |  |
| Yes | 2.545 (1.609 - 4.163) | 0.0001 | 2.347 (1.285 - 4.581) | 0.0079 |
| **Baseline B2 Microglobulin at LD** |  |  |  |  |
| <5.5 | 1.00 (Ref) |  | 1.00 (Ref) |  |
| ≥5.5 | 1.343 (0.7549 - 2.315) | 0.2988 | 1.695 (0.7786 - 3.510) | 0.1648 |

**Abbreviations:** EMD, extramedullary disease; ECOG PS, Eastern Cooperative Oncology Group Performance Status; R-ISS, Revised International Staging System; FISH, fluorescence in-situ hybridization; SCT, stem cell transplantation; CRP, C-reactive protein; LDH, lactate dehydrogenase; CrCl, creatinine clearance; LVEF, left ventricular ejection fraction
